# A bibliometric analysis of the research status and trends in studies on polymyositis and dermatomyositis with interstitial lung disease from 2000 to 2022 using Web of Science

**DOI:** 10.1002/iid3.1190

**Published:** 2024-02-20

**Authors:** Xiao‐Na Ma, Wei Feng, Shu‐Lin Chen, Xiao‐Qin Zhong, Xue‐Xia Zheng, Chang‐Song Lin, Qiang Xu

**Affiliations:** ^1^ State Key Laboratory of Traditional Chinese Medicine Syndrome The First Clinical Medical College of Guangzhou University of Chinese Medicine Guangzhou Guangdong China; ^2^ Department of Rheumatology Guangzhou University of Traditional Chinese Medicine of the First Affiliated Hospital Guangzhou Guangdong China

**Keywords:** bibliometric analysis, hotspots, interstitial lung disease, polymyositis/dermatomyositis, research trend

## Abstract

**Background:**

The main subtypes of idiopathic inflammatory myopathies (IIMs)—polymyositis (PM) and dermatomyositis (DM)—are often presented as interstitial lung disease (ILD) in clinical practice; therefore, many researchers have combined the three studies into PM/DM with ILD.

**Methods:**

Using bibliometrics, the research status, progress, and hotspots of PM/DM with ILD between 2000 and 2022 were studied. Literature data on PM/DM with ILD were retrieved from the Web of Science (WoS) database for the research period. Visualization software, including VOSviewer, Pajek, CiteSpace, and Scimago Graphica were used for bibliometric analysis.

**Results:**

A total of 1555 relevant articles were obtained, and the overall research in this field showed an increasing trend. Regarding contributing countries and venues, Japan published the most articles while *Rheumatology* was the most prolific journal. Regarding authors, the most published article was by Wang Guochun from Changchun University of Technology in China. Keyword analysis and cocited literature cluster analysis showed that diagnosis, classification, autoantibodies, antibodies, prognosis, complications, and treatment of PM/DM with ILD have been hot topics in this field recently. Moreover, our study shows that anti‐mda5 antibody, mortality, gene 5 antibody, IIMs, double‐blind, and prognostic factors, among others, may be new hot topics.

**Conclusion:**

This study found that research on PM/DM with ILD has increased over time, and scholars are paying more attention to this field. The development of new drugs for the management, treatment, and prevention of PM/DM with ILD is the primary task of researchers and a direction for future research in this field.

## Graphical abstract

Based on the Web of Science database, the current status and trends of research on polymyositis (PM)/dermatomyositis (DM) with interstitial lung disease (ILD) (2000−2022) were analyzed in terms of annual publications, authors, institutions, countries, and keywords. The study found that researchers' interest in PM/DM with ILD has been increasing year by year, and the diagnosis, classification, autoantibodies, antibodies, prognosis, complications, and treatment of PM/DM with ILD have been a hot topic in the field. Anti‐mda5 antibody, mortality, gene 5 antibody, idiopathic inflammatory myopathies, double‐blind, and prognostic factors, among others, may be new hot topics.

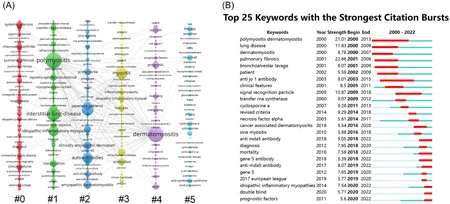

## 1 INTRODUCTION

Idiopathic inflammatory myopathies (IIMs), whose main subtypes are polymyositis (PM) and dermatomyositis (DM), are a group of multiple autoimmune diseases characterized by muscle involvement and extramuscular manifestations, including skin and lung manifestations.^1^, ^2^, ^3^ Patients with PM/DM often present with interstitial lung disease (ILD), a common complication of IIMs that mainly involves the lung and is the most important prognostic factor for death in patients with PM and DM.^4^ The global prevalence of ILD in PM/DM patients is approximately 41%.^2^, ^5^ In addition to the clinical manifestations of PM/DM associated with muscle inflammation and extramuscular manifestations, patients with PM/DM combined with ILD may suffer acute respiratory symptoms within 1 month of onset, worsening of radiographic interstitial changes, and progressive dyspnea and hypoxemia.^2^, ^6^ A pulmonary function examination in patients with PM/DM is important for the diagnosis of ILD and for monitoring disease progression over time.^7^ Therapeutically, the proper management of ILD is important for improving the PM/DM patient outcomes. ILD activity and severity depend on the disease subtype, and clinicians should determine treatment strategies based on the disease subtype in each PM/DM patient.^8^ There is no standard treatment for the management of PM/DM with ILD, and practices vary widely. Corticosteroids are the first‐line treatment for myositis‐associated ILD; however, other immunosuppressive treatments are often used.^7^

Bibliometrics is a quantitative science field that uses citation analysis and other methods to evaluate research performance and is a mature method for the quantitative evaluation of academic productivity.^9^, ^10^ Bibliometric analysis can be used to identify influential research that has shaped medical practice and nurtured new research ideas.^9^, ^11^ Bibliometrics can quantify the relationships among high‐yield authors, countries and institutions, journals, and keywords, thereby revealing new technologies, hotspots, and trends in the various fields of clinical medicine.^12^ Recently, the application of bibliometrics has greatly increased due to the improvement of scientific research and thus the increasing number of publications in different fields. In the process of scientific research, understanding the relevant knowledge and predicting and studying new research directions is essential, and bibliometrics has provided such opportunity and convenience for researchers. Currently, bibliometrics has been widely used in the fields of stomatology, ophthalmology, nephrology, neurology, tissue psychology, and autoimmune diseases.^13^, ^14^, ^15^, ^16^, ^17^, ^18^

In the period from 2000 to 2022, studies on PM/DM with ILD have been increasing. These studies include the clinical characteristics, diagnosis, treatment, specific antibodies, autoantibodies, complications, risk factors, and classification of PM/DM with ILD. Though researchers are paying increasing attention to this field, it is unclear how much research is being conducted. Through the use of bibliometrics, this study analyzes the research status, progress, directions, hotspots, and other aspects in this field over the past 20 years. The aim is to provide information channels for researchers who want to know the specific status of related study areas, predict upcoming hotspots, and provide references for them to select future research directions.

## 2 MATERIALS AND METHODS

### 2.1 Data sources and rearch strategies

**Figure 1:**
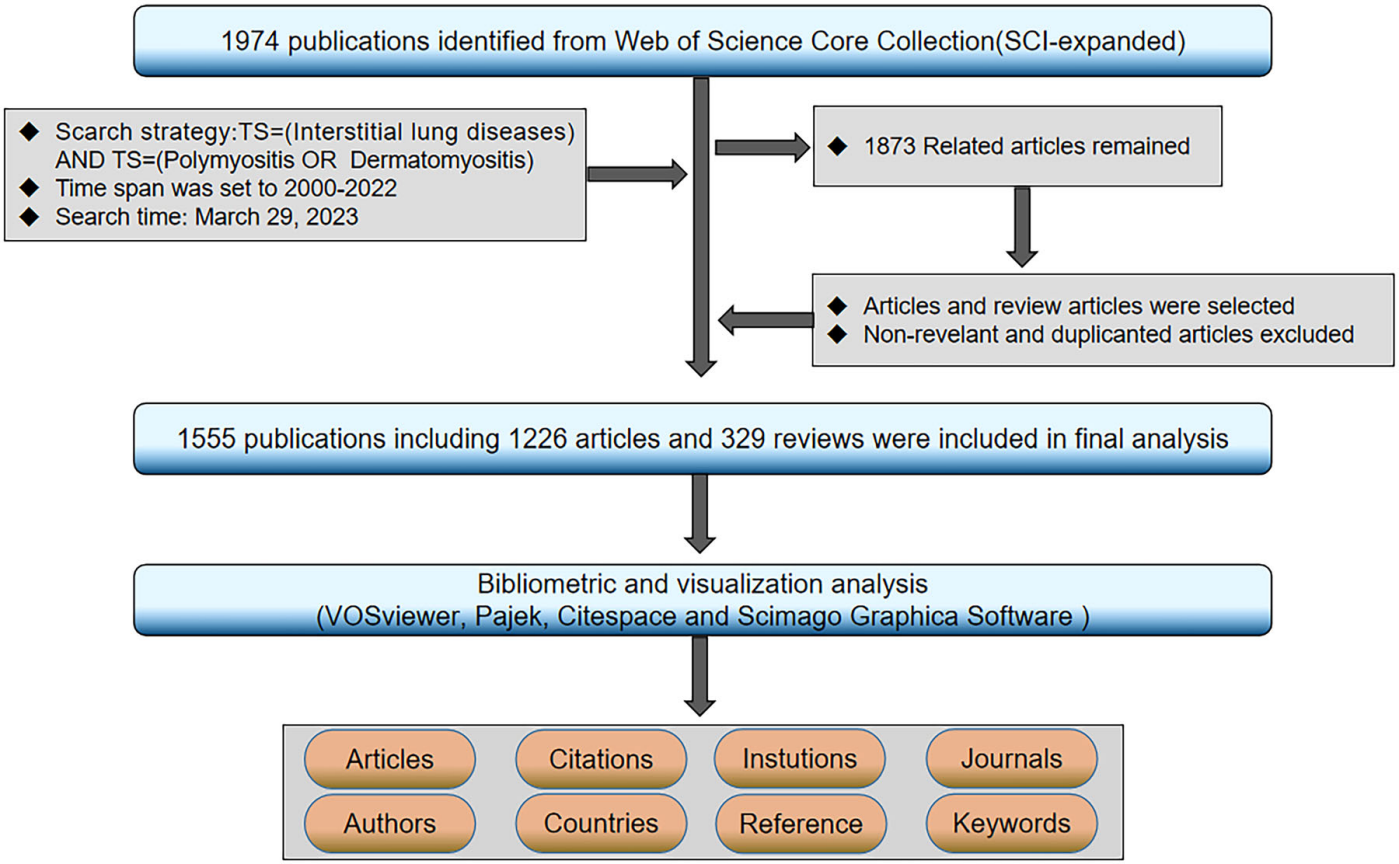
Flowchart of the literature search, screening, and analysis.

In this paper, the data collected from the Science Citation Index Expanded in the Web of Science (WoS) database, and through the library of Guangzhou university of Chinese medicine in south China's Guangdong province for a visit. WoS database is one of the world's major repositories of scientific results, which covers a large number of high quality academic journals and a comprehensive coverage of key research results from around the world, and its resources are considered to hold some of the most cited references.^19^, ^20^ WoS Core Collection (WoSCC) database is a typical citation database, which contains abstracts and other relevant data, such as citation and collaborative information, and is convenient for bibliometric analysis. Moreover, it can directly provide references conforming to specific format requirements stipulated by document metrology software.^21^ Since PM, DM and ILD are the subjects of our study, we conducted a search for these subject words to ensure that the data obtained are closely related to this study. Specific inclusion and exclusion criteria are shown in Figure 1. The data retrieval took place on March 29, 2023, using the following retrieval strategies: (TS = [ILD] AND [TS = PM OR DM]). A total of 1974 articles were obtained, of which 1873 were obtained after excluding 101 nonrelevant articles. Then original articles and retrospective articles were selected, and after excluding relevant and duplicate entries, a total of 1555 final valid publications were selected for further analysis as the final data set.

### 2.2 Data extraction

The extraction of relevant data was completed by three researchers (X. N. M., W. F., and S. L. C.), respectively. During the extraction process, the data were constantly compared. If there were differences in the data, targeted discussions were made until the results were obtained and a consensus was reached, so as to ensure the authenticity and accuracy of the extracted data. Finally, the authors, titles, countries, journals, year of publications, number of citations, highly cited literatures, institutions, keywords, abstracts, and references are recorded. At the same time, two researchers and the third researcher together classified the main categories of the articles into diseases, etiology and pathogenesis, clinical features, diagnosis, treatment, prevention, immunity, autoantibodies, classification, and so forth. Hirsch index (H‐index) was obtained by the WoS database. Journal information including impact factor (IF) and category (Q1−Q4) quartiles was collected from the 2022 Journal Citation report.^21^

### 2.3 Data analysis

The results of the bibliometric analysis were analyzed and statistically plotted using Microsoft Excel 2019 (Microsoft Corporation). Global PM/DM with ILD and annual trends in the top 10 countries were evaluated by linear regression analysis from 2000 to 2022. Microsoft Excel 2019 and Microsoft Word 2019 (Microsoft Corporation) were used to analyze the number of publications, total citations, average citations, H‐index, and other indicators from various aspects such as countries, institutions, and journals. Classification data were expressed as count (percentage), and *p* < .05 was considered statistically significant. Visual software such as VOSviewer, CiteSpace, Pajek, and Scimago Graphica were used for bibliometric analysis. The global distribution of national publications and national collaborative networks are mapped using Scimago Graphica software.Use VOSviewer and Pajek software to visualize cooccurrence analysis of institutions and authors. In the visualization, the colors represent the clusters to which journals are assigned by using clustering techniques, with different clusters corresponding to different areas of study. Nodes represent institutions or authors, the lines between nodes represent cooperative relationships,^19^, ^22^ the area of nodes represents the number of nodes, and the thickness of lines between nodes represents the intensity of cooperation.

## 3 RESULTS

### 3.1 Articles and citation trends

**Figure 2:**
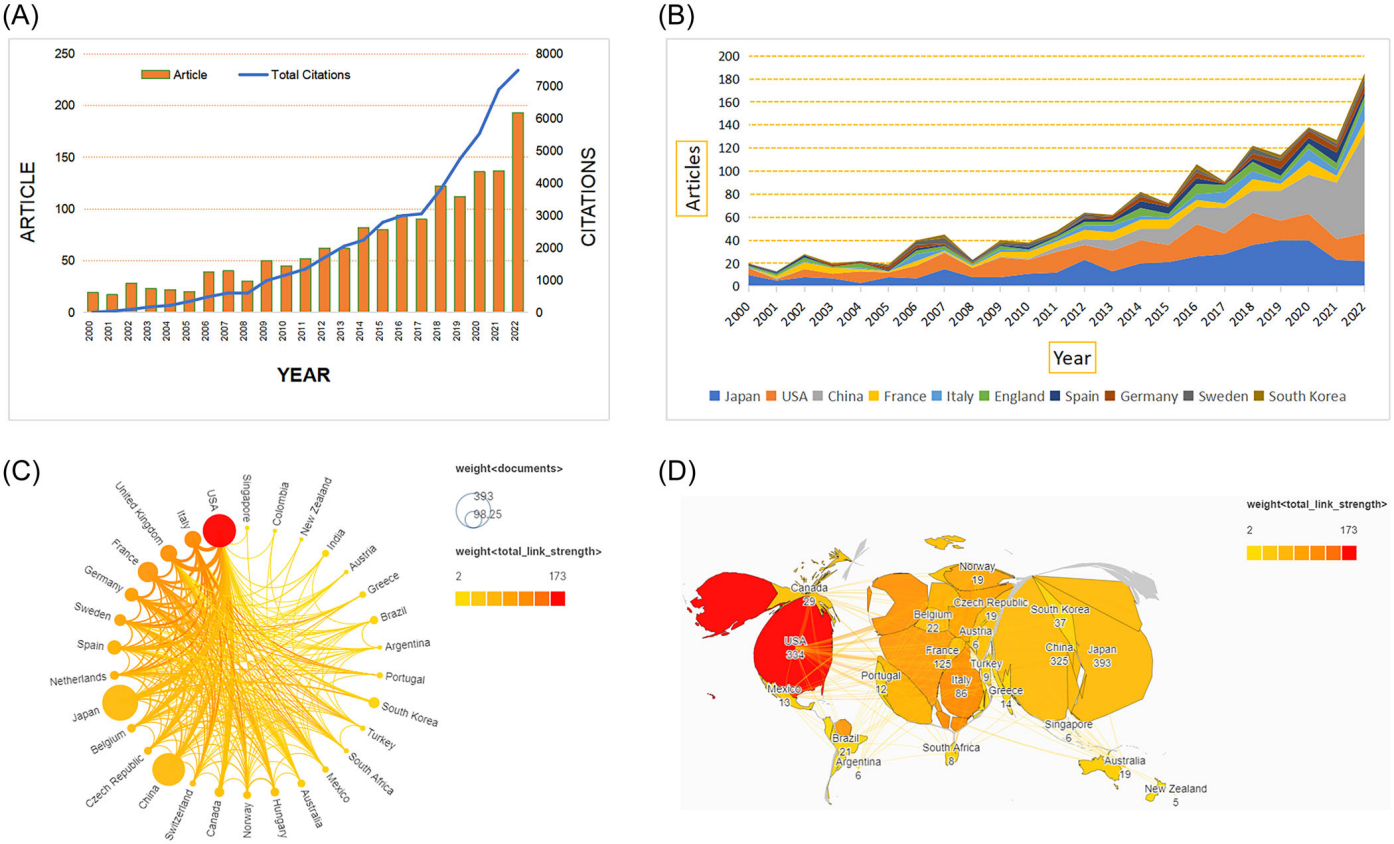
(A) Trends in annual citations and publications from 2000 to 2022. (B) Annual trends in the number of publications in the top 10 countries. (C) Cooperation network among countries. (D) A world map of countries' contributions, which shows the number of publications for each country.

Through screening of the literature data, 1555 final valid publications were selected, including 1226 original articles and 329 retrospective articles. The distribution of annual research publications on PM/DM with ILD is shown in Figure 2A. From 2000 to 2022, the number of publications related to PM/DM with ILD increased and then decreased each year; however, an overall increasing trend was observed, and the number of publications reached 193 in 2022. The number of citations totaled 49,310, with a total of 31,081 citations, excluding self‐citations (18,229). The average number of citations of each publication was 32.32, showing a linear growth trend (*R* ^2^ = 0.8535). The growth trend was most significant during the 2017−2022 period, reaching its peak in 2022, at 7491 times.

### 3.2 Global research status

#### 3.2.1 Country analysis

As shown in Figure 2B, Japan and the United States dominated the number of published articles in this field compared to other countries, although there are some fluctuations. From 2014 to 2020, the number of published articles in Japan continued to increase, and from 2017 to 2020, Japan ranked first in the number of published articles annually. Meanwhile, Japan's annual publication volume remained at 40 in both 2019 and 2020, making it the largest contributor in this field. It is also worth mentioning that China, which ranks third, has maintained a general continuous growth trend since 2010. By 2021, China's annual publication volume had exceeded Japan and the United States, at 49, while in 2022, the number of articles published annually reached 87.

Figure 2C shows a chord diagram of the international cooperation network among different countries. The connecting lines in the figure represent international cooperation between countries; the thicker the lines, the closer the cooperation. Node size represents the number of published papers; the larger the node, the more papers published in that country. Darker colored nodes indicate the number of cooperative relationships between countries; the darker the node color, the more cooperation with other countries. The United States leads in research cooperation with other countries, at 98.25, and has the closest cooperation with Japan, Canada, Ukraine, Italy, Spain, and China, among others.

From the chord diagram of national cooperation (Figure 2C) and the world map of national contributions (Figure 2D), it is evident that Asia, North America, Europe, and other regions have the largest number of published articles, with Japan, the United States, China, and France leading. Table 1 shows the results of our collation of publications in the top 10 countries with the most prominent contributions. The table shows that Japan had the most published articles with 393, and its total number of citations (14,089) and H‐index (64) also ranked second. However, Japan ranked far behind in citation average (35.85). The United States was temporarily ranked second with 334 published articles; however, its total number of citations (18,947) and H‐index (74) surpassed that of Japan, ranking first. In addition, its citation average was 56.73, surpassing the United Kingdom (54.39; third) and Sweden (58.7; first). In publications, China ranked third, with 298 articles. Its total number of citations decreased significantly compared to Japan and the United States, but still ranked third at 4726, while its average number of citations and H‐index were relatively lower.

**Table 1:** Top 10 countries with the most articles.

Rank	Country	No of papers	Total citation	Average citation	H‐idex
1	Japan	393	14,089	35.85	64
2	USA	334	18,947	56.73	74
3	China	325	4726	14.54	33
4	France	125	4722	37.78	41
5	Italy	86	3007	34.97	30
6	England	84	4569	54.39	38
7	Spain	62	1913	30.85	22
8	Germany	57	2604	45.68	23
9	Sweden	43	2524	58.7	25
10	South Korea	37	1308	35.35	16

#### 3.2.2 Journal analysis

Studies on PM/DM with ILD have been published in over 300 journals. Table 2 shows an analysis of the top 10 journals with the most publications in the field. *Rheumatology* had the highest number of publications (103; 6.62%), followed by *Clinical Rheumatology* (83: 5.34%; second) and *Internal Medicine* (47; 3.02%; third). Regarding IF, *Rheumatology* not only published the most articles but also had the highest IF, at 7.046. This was followed by Treatment of Arthritis Recurrence (IF = 5.606) and the *Journal of Rheumatology* (IF = 5.346). Of the top 10 journals, only *Rheumatology* and Treatment of Arthritis Relapse were included in Q1. It is worth noting that most of these stages (60%) were classified as rheumatology, indicating that studies related to PM/DM with ILD are highly valued by researchers in the field of rheumatology. Regarding country distribution, four of the top 10 journals are from the United Kingdom, two are from Japan, and the others are from the United States, China, Germany, and Italy. Notably, most of the active journals are located in either Europe or Asia.

**Table 2:** Top 10 journals contributing to publications.

Rank	Journal	No of papers (%)	Impact factor (2022)	Quartile in category
1	*Rheumatology*	103 (6.62)	7.046	Q1
2	*Clinical Rheumatology*	83 (5.34)	3.65	Q3
3	*Internal Medicine*	47 (3.02)	1.282	Q4
4	*Modern Rheumatology*	46 (2.96)	2.862	Q4
5	*Journal of Rheumatology*	45 (2.90)	5.346	Q2
6	*Clinical and Experimental Rheumatology*	44 (2.83)	4.862	Q2
7	*Rheumatology International*	43 (2.77)	3.58	Q3
8	*Respiratory Medicine*	39 (2.51)	4.582	Q2
9	*Current Opinion In Rheumatology*	32 (2.06)	4.941	Q2
10	*Arthritis Research Therapy*	27 (1.74)	5.606	Q1

#### 3.2.3 Institution analysis

**Figure 3:**
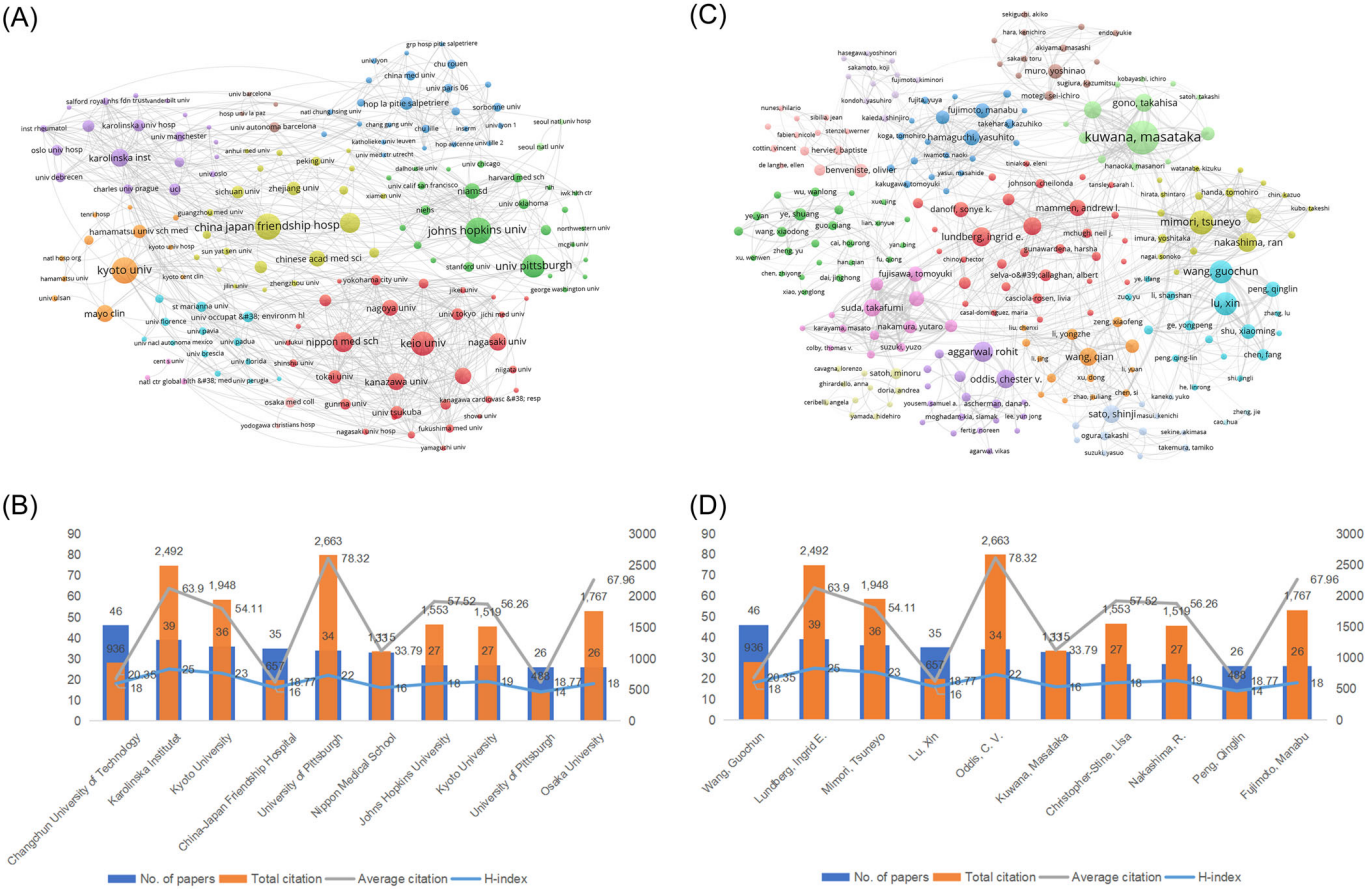
(A) Cooperation network among institutions. (B) The publication counts, citations, and H‐index of the top 10 prolific institutions. (C) Cooperation network among authors. (D) The publication counts, citations, and H‐index of the top 10 prolific authors.

This study found more than 2500 organizations that contributed to the field. Figure 3A shows the complex cooperative relationships among these institutions, and Supporting Information S1: Figure 1A shows their average annual cooperative network. Cooperation between institutions is scattered across Asia, North America, Europe, and other developed or developing countries. The frequent cooperation between institutions such as Kelouniv, Johns Hopkins University, and the China−Japan Friendship Hospital show the important and influential role played by these institutions in the field. In addition, a number of relatively new institutions have emerged in the PM/DM with ILD research space, including Osaka Medical College, Seoul National University, and Universitat Autònoma Barcelona, among others.

Figure 3B and Table 3 shows the top 10 most influential interagency cooperation, four of which are from Japan, three from the United States, two from China, and one from Sweden. Among them, Changchun University of Technology in China ranked first, with 46 papers published; however, its total citation frequency, average citation rate, and H‐index ranked relatively low among the 10 universities, indicating that the quality of its papers should be further improved. Regarding total citations and average citation rate, the University of Pittsburgh ranked first, with 2663 citations and an average citation rate of 78.32, although it ranked third in H‐index (22). Sweden's Karolinska Institute ranked first in H‐index, at 25, though it ranked second in number of published articles (39) and total citations (2492) and third on average citation rate (63.9). Osaka University ranked second on average citations (67.96), and Kyoto University ranked third in number of publications (36) and total citations (1948), and second in H‐index (23).

**Table 3:** Top 10 most prolific authors and institutions on polymyositis/dermatomyositis with interstitial lung disease research.

Rank	Author	No of papers	Total citation	Average citation	H‐index	Institution	Country
1	Wang, Guochun	46	936	20.35	18	Changchun University of Technology	China
2	Lundberg, Ingrid E.	39	2492	63.9	25	Karolinska Institutet	Sweden
3	Mimori, Tsuneyo	36	1948	54.11	23	Kyoto University	Japan
5	Lu, Xin	35	657	18.77	16	China‐Japan Friendship Hospital	China
4	Oddis, C. V.	34	2663	78.32	22	University of Pittsburgh	USA
6	Kuwana, Masataka	33	1115	33.79	16	Nippon Medical School	Japan
7	Christopher‐Stine, Lisa	27	1553	57.52	18	Johns Hopkins University	USA
8	Nakashima, R.	27	1519	56.26	19	Kyoto University	Japan
9	Peng, Qinglin	26	488	18.77	14	University of Pittsburgh	USA
10	Fujimoto, Manabu	26	1767	67.96	18	Osaka University	Japan

### 3.3 Representative authors and articles

#### 3.3.1 Authors analysis

Figure 3C and Supporting Information S1: Figure 1B show the author collaborative visualization network and that for the average year, respectively. Combined with the collation and quantification of the analysis results of the top 10 productive authors (Figure 3D, Table 3), the following findings can be highlighted. Among the top 10 authors, Ingrid Lundberg (Sweden), Masataka Kuwana (Japan), Guochun Wang (China), and Tsuneyo Mimori (Japan) were most frequently at the center of cooperation. Among the top 10 authors with the most published articles, Wang (Changchun University of Technology) was the author with the most published articles at 46, followed by Lundberg (39) and Mimori (36). Among these three, Lundberg (25) had the highest H‐index score. Mimori and Nakashima are from Kyoto University, while Chester Oddis and Qinglin Peng are from the University of Pittsburgh. Among the top 10 authors' institutions, four were in Japan, three in the United States, two in China, and one in Sweden. Clearly, these top authors have considerable influence in the field. Moreover, Cao Hua, Jie Zheng, and Koji Sakamoto are relatively new but active authors in the field.

#### 3.3.2 Analysis of highly cited literature

**Figure 4:**
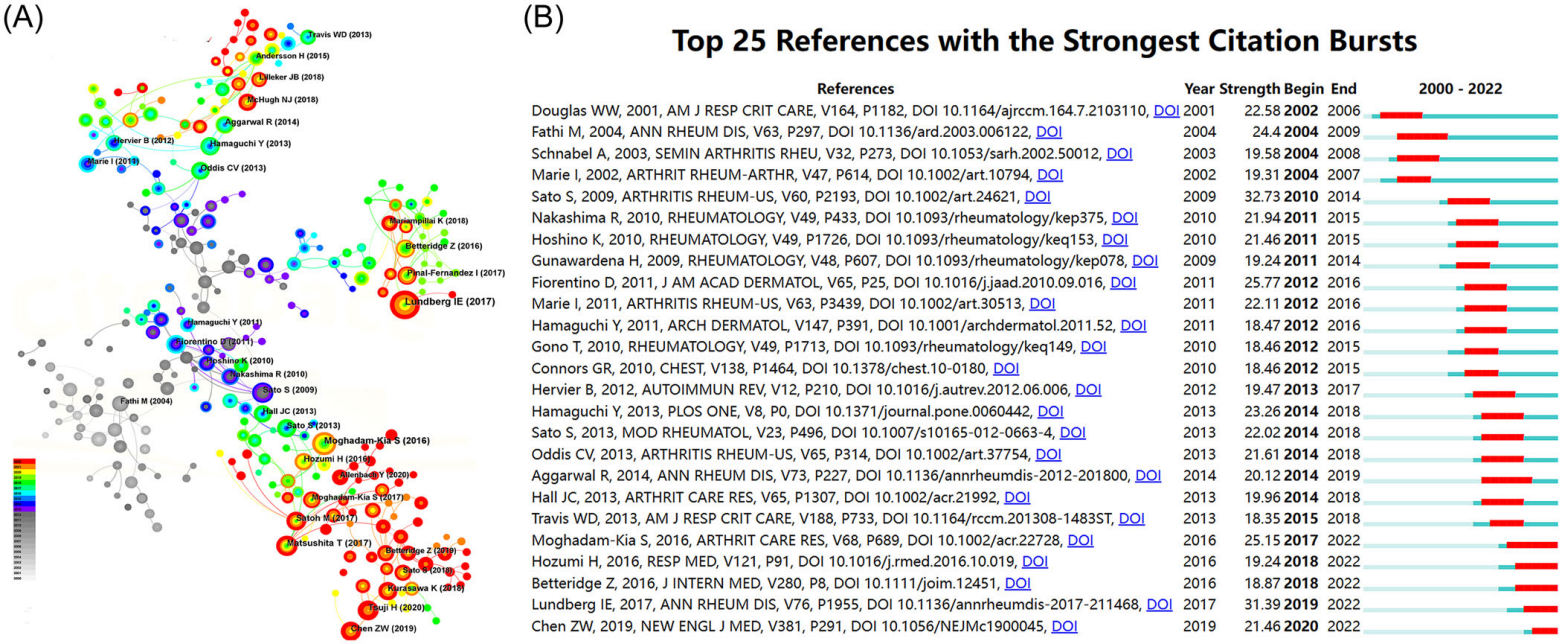
(A) The knowledge map of cocited reference. (B) The top 25 references with the strongest citation bursts.

The top 10 articles cited in the past 20 years are summarized in Table 4, with the maximum number of citations reaching 1232 and the minimum at 294. The top 10 articles include eight original articles, one retrospective study, and one systematic review. Three were published in *Arthritis and Rheumatism* (IF = 8.955), two in the *American Journal of Respiratory and Critical Care Medicine* (IF = 30.528), two in the *Journal of the American Academy of Dermatology* (IF = 15.487), one in *Lancet* (IF = 202.731), and two in the *Journal of the American Academy of Dermatology* (IF = 15.487). One paper was published in the *European Respiratory Journal* (IF = 33.795), and the first paper with 1232 citations was published in *Immunity* (IF = 43.474). Figure 4A shows the knowledge map of highly cocited literature from 2000 to 2022.

**Table 4:** Top 10 high‐cited articles on polymyositis/dermatomyositis with interstitial lung disease research.

Rank	Authors	Year	Article title	Journal	Total citations
1	Loo et al.	2011	Immune signaling by RIG‐I‐like receptors	*Immunity*	1232
2	Dalakas et al.	2003	Polymyositis and dermatomyositis	*Lancet*	967
3	Bouros et al.	2002	Histopathologic subsets of fibrosing alveolitis in patients with systemic sclerosis and their relationship to outcome	*American Journal of Respiratory and Critical Care Medicine*	533
4	Sato et al.	2005	Autoantibodies to a 140 kd polypeptide, CADM‐140, in Japanese patients with clinically amyopathic dermatomyositis	*Arthritis and Rheumatism*	449
5	Sato et al.	2009	RNA helicase encoded by melanoma differentiation‐associated gene 5 is a major autoantigen in patients with clinically amyopathic dermatomyositis association with rapidly progressive interstitial lung disease	*Arthritis and Rheumatism*	403
6	Douglas et al.	2001	Polymyositis‐dermatomyositis‐associated interstitial lung disease	*American Journal of Respiratory and Critical Care Medicine*	377
7	Kim et al.	2010	Usual interstitial pneumonia in rheumatoid arthritis‐associated interstitial lung disease	*European Respiratory Journal*	351
8	Fiorentino et al.	2011	The mucocutaneous and systemic phenotype of dermatomyositis patients with antibodies to MDA5 (CADM‐140): a retrospective study	*Journal of the American Academy of Dermatology*	344
9	Marie et al.	2002	Interstitial lung disease in polymyositis and dermatomyositis	A*rthritis and Rheumatism*	311
10	Gerami et al.	2006	A systematic review of adult‐onset clinically amyopathic dermatomyositis (dermatomyositis sine myositis): a missing link within the spectrum of the idiopathic inflammatory myopathies	*Journal of the American Academy of Dermatology*	294

### 3.4 Review of research hotspots and frontiers

#### 3.4.1 Cluster analysis of cocited literature

The literature cocitation network diagram in Figure 4A was divided into 20 clusters (Supporting Information S1: Figure 2). Because the logarithmic likelihood can better cover the “uniqueness and coverage” of all labels, these clusters were labeled by extracting terms from the titles of the referenced publications.^21^, ^23^, ^24^ The #0 autopsy study was the largest cluster, followed by #2 antisynthetase syndrome and #3 clinical association. Figure 4B shows the top 25 references with the strongest citation bursts, among which the top three were Douglas et al., Fathi et al., and Schnabel et al.^4^, ^25^, ^26^

#### 3.4.2 Keywords analysis

**Figure 5:**
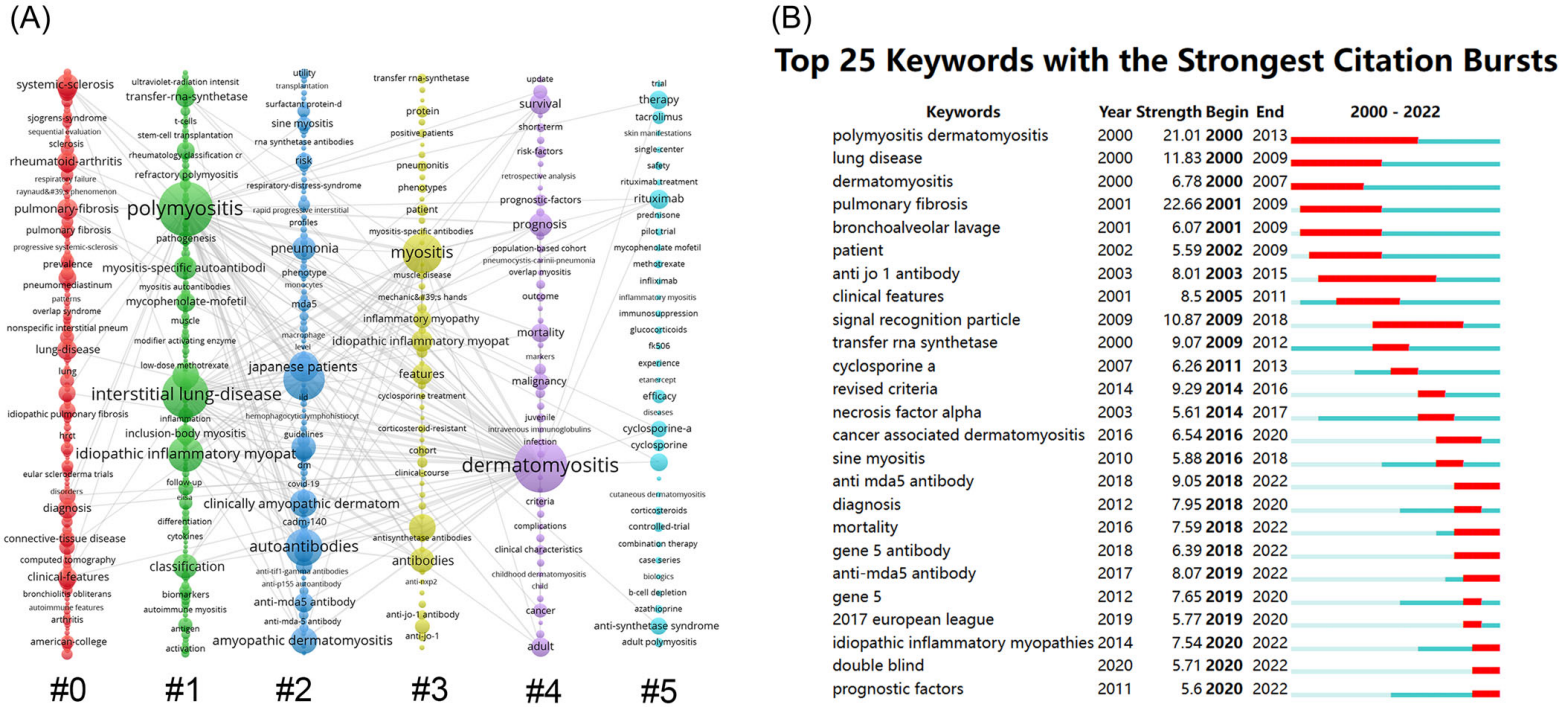
(A) Cooccurrence network visualization of keywords, where each column represents a set of studies generated by VOSviewer. (B) The top 25 keywords with the strongest citation bursts.

Figure 5A shows the visualization of keywords that appear more than five times simultaneously in this field of research. The size of the node is proportional to the frequency of keyword occurrence; the thicker the line between two nodes, the higher the frequency of their simultaneous occurrence.^21^, ^27^, ^28^ The keywords were divided into six groups, according to their specific content as follows: #0 diagnosis and lung disease, #1 classification, #2 autoantibodies, #3 antibodies, #4 prognosis and complications, and #5 treatment. Supporting Information S1: Figure 3A shows the average year visualization of the keywords stacked in chronological order. The purple−yellow gradient in the time trajectory corresponds to the average number of keyword occurrences from 2010 to 2020. Among these keywords, anti mda5 antibody, prognostic factors, and IIMs have been the main research themes in recent years. We screened and visualized the top 25 keywords with the largest number of keyword cooccurrences (Supporting Information S1: Figure 3B). Meanwhile, through the keyword breakout analysis in Figure 5B, we found that the top 25 keywords with the strongest citation bursts that continued through 2022 include anti mda5 antibody (anti‐mda5 antibody), mortality, gene 5 antibody, IIMs, double blind, and prognostic factors. These results also confirm that the preceding keywords are new research topics in recent years, and their research interest is likely to increase over the next few years.

## 4 DISCUSSION

This study presents a bibliometric analysis of studies on PM/DM with ILD from 2000 to 2022 using bibliometric analysis methods and knowledge map visualization software. The knowledge distribution characteristics and emerging trends of PM/DM with ILD were presented at the macro‐ and micro‐levels,^29^ making the entire knowledge field clearer, more intuitive, and systematic, and providing access to the in‐depth understanding and research in this field. It also provides ideas for predicting future research hotspots. Based on bibliometric methods, we analyzed 1555 papers published in the WoS database on PM/DM with ILD studies, including annual publication trends and national, institutional, and authorial collaboration. The relationship between the number of publications and journal, citations, influence factor, H‐index, and other factors was analyzed. Therefore, this study forecasts that anti mda5 antibody (anti‐mda5 antibody), mortality, gene 5 antibody, IIMs double‐blind, and prognostic factors are likely to be the next hot topics. Research on PM/DM with ILD has been growing over the past 20 years, which may be closely related to the increasing incidence and clinical frequency of this field as well as researchers' interest and focus. In 2022, in particular, the number of publications in this field were far higher than in any other previous year. Evidently, the popularity of research in this field reached an unprecedented new level in 2022, indicating an inevitable increasing trend in research interest and attention in this field.

This significant growth trend may be related to changes in the national population and increase in national economic income. Improvements in the national economic level and increases in population are closely correlated with the number of publications in this field.^19^, ^21^ We found that almost all major contributors to research in this field are high‐income countries, except for China, which is an upper‐middle‐income country.

In terms of IF and total citations, the top three countries are the United States, Japan, and France, respectively, whose economic strength is widely recognized by the international community. Evidently, strong national economic strength and improvement in the national development level may offer a solid foundation for scientific research because these factors directly affect the scientific research funding level and researchers' English fluency, in addition to the country's Internet penetration rate. The combined effects of these factors have led to an uneven number of publications in this field.^19^, ^30^, ^31^

National economic condition may also be an important factor affecting the frequency of cooperation between countries. The higher the level of national development, the more opportunities for communication and cooperation, leading to access to more platforms, broader scientific research fields, richer and more in‐depth and detailed scientific research content, and higher quantity and quality of published articles.^32^, ^33^ As shown in the visualization network diagram of national cooperation, the United States has a relatively high number of international cooperation with other high‐ or middle‐income countries such as France, Japan, and China.

From the literature data in our study, we found that the studies include considerable research on the clinical manifestations, pathogenesis, risk factors, treatment, specific autoantibodies, clinical and daily management, and complications of related diseases in this field. ILD increases the mortality rate in patients with PM/DM. Meanwhile, in our keyword analysis, PM/DM with ILD as an autoimmune disease can be complicated by rheumatoid arthritis, systemic lupus erythematosus, and other immune system‐related diseases. Notably, it is also associated with the occurrence of a variety of malignant tumors.^19^, ^34^ Therefore, as the primary clinical feature of IIMs, the search for autospecific antibodies is important to intervention in the development of PM/DM with ILD. The study of specific antibodies in this field and the formulation of treatment plans are of great significance for patients with PM/DM with ILD, to reduce their symptoms, improve their quality of life, reduce the incidence and mortality of the disease, and should become a popular research direction for researchers.

Our study included 340 journals, the most representative of which were *Rheumatology*, *Clinical Rheumatology*, *Internal Medicine*, *Modern Rheumatology*, and *Clinical and Experimental Rheumatology*. Among them, *Rheumatology* and *Clinical Rheumatology* from the UK ranked first and second, while *Internal Medicine* and *Modern Rheumatology* from Japan ranked third and fourth, respectively. *Clinical and Experimental Rheumatology* from Italy ranked fifth. These journals are leading the way in their respective countries. This may be related to the tendency of journals to publish papers, and authors to contribute to journals in their own region.^19^ Regarding the content and research direction of the articles published in these active journals, their driving force and influence on the relevant hot topics in this field dominate, which shows the unified research results for researchers in this field, indicating that the latest scientific research direction and entry point guidance provides the basis and platform and plays an important role in driving the research upsurge in this field.

This study analyzed the citation counts of the articles. Among the top 10 articles with the most citations listed, the one with the most citations was an article published by Loo et al., which mainly studied RIG‐I‐like receptor (RLR)‐mediated immune signal transduction. The study suggests that abnormal RLR signaling, or dysregulation of RLR expression, is associated with the development of autoimmune diseases, and understanding RLR signaling and response will provide insights to guide RLR‐targeted therapy in antiviral and immunomodification applications.^35^ Thus, it is evident that research in this field focuses on the mechanisms of immune regulation, infection control, and inflammation control of the receptor autoantibodies of autoimmune diseases to find targeted therapies. The second most cited article was published by Dalakas on PM and DM, focusing on the clinical symptoms, diagnosis, treatment, and prognosis of IIMs, indicating that people are enthusiastic about gaining a comprehensive understanding of IIMs. Notably, this study also aimed to identify new immunomodulators for the treatment of difficult cases of IIMs. Thus, it once again calls for the key treatment of related autoimmune diseases, such as IIMs, to identify pathogenic autoantigens that play the immunomodulatory role of autospecific antibodies.^36^

Among the top 10 most‐cited articles, the antibodies discussed include RLRs,^35^ MHCI antigen‐associated antibodies,^36^ 140 kd polypeptide CADM‐140 autoantibodies,^37^ MDA5 antibodies (CADM‐140),^38^, ^39^ Jo‐1 antibodies,^25^, ^40^ antinuclear antibodies, Jo‐2 antibodies, and Mi‐3 antibodies.^41^ We noted that there were two articles on the DM‐140 and Jo‐1 antibodies of CA DM, respectively, which were published by authors with considerable citations and great influence. This indicates that the DM‐140 and Jo‐1 antibodies of CA DM attracted more research attention in the study of autospecific antibodies in this field. They also appear to play an important role in the research of myositis‐related diseases. Nearly 10 types of antibodies related to myositis have been mentioned in influential articles; other antibodies mentioned in articles outside this research field include anti‐RO52 antibody,^42^ anti‐NXp2 antibody,^43^ and anti‐SAE antibody,^44^ which also play an important guiding role in the interventionist treatment of myositis. The presence of myositis‐specific autoantibodies usually manifests as varying degrees of skin or muscle inflammation, and these autoantibodies help inform the diagnosis, management, and prognosis of the disease.^44^ The citation frequency of PM/DM with ILD has increased significantly in recent years; however, the citation frequency of studies on autoantibodies and specific antibodies has not reached a leading state. Therefore, this study considers that research in this field needs to be popularized and in‐depth. Such change may provide ideas and innovation opportunities to find channels and methods to treat myositis‐related diseases and improve our medical technology level in this field to a greater extent, achieving satisfactory clinical efficacy.

In the keyword cooccurrence network visualization analysis (Figure 5A), the occurrence times of PM (green), DM (purple), and ILD (green) ranked in the top three successively, which was consistent with our search theme and focus. Myositis, autoantibodies, pulmonary diseases (such as pneumonia and pulmonary fibrosis), idiopathic inflammatory myoocular carcinoma, rheumatoid arthritis, systemic lupus erythematosus, and other keywords appeared relatively more frequently. Pulmonary, rheumatoid, and ocular diseases were shown to be associated with PM/DM with ILD.^19^ We also noticed that the occurrence of malignant tumors was not low in frequency. The occurrence of malignant tumors affects the mortality rate of related diseases in this field. Researching the connection and evolution process between the two and the possible intervention measures is an effective way to reduce the incidence rate of malignant tumors in this field, and thus reduce the mortality rate of related diseases. This may also become a new hot topic in this field. What's more, combining the keywords and citation burst analysis, we found that researchers gradually shifted from the study of the clinical characteristics of diseases such as PM, DM, and ILD to the study of the diagnosis, treatment, and mortality of the diseases, especially for the study of the anti mda5 antibody, gene 5 antibody, and anti‐mda5 antibody, which has been continued to 2022, which once again proves that the study of the auto‐specific antibody‐related research may be the new research hot topic and inevitable trend in recent years.

The top three authors in terms of publication volume—Wang, Lundberg, and Mimori—came from China, Sweden, and Japan, respectively, and all three countries were also among the top 10 contributors to the study. Moreover, the number of citations and H‐index of Lundberg and Mimori ranked second and third, respectively, among the top 10 authors. This may reflect the research capacity of the author's corresponding institution or university, the frequency of cooperation between institutions, and the availability of research funds. This study also noted that Oddis from the United States, ranked first in terms of total and average citation times but ranked third in H‐index. It is difficult to avoid the conclusion that the level of scientific research is closely related to a country's economic status and development level, which may also affect the research abilities of various research teams and the frequency of international cooperation. Research teams and institutions with numerous strengths and outstanding contributions to the field are influenced by several factors, which may be directly related to the country's high economic development.

Considering that WoS is an English database, all the literature included in our research was in English, which indicates that the influence of the native language of different countries may also be a limitation for some nonnative English speakers. From the top 10 countries in publication volume, only the United States and the United Kingdom are English‐speaking; however, the remaining eight countries have a high English penetration rate. From this perspective, language may also impact the field of study.

## 5 CONCLUSIONS

We studied and analyzed published articles on PM/DM with ILD from 2000 to 2022 and found that the volume of articles published in this field has generally shown an increasing trend over time, with the main source countries being high‐income countries. As an upper‐middle‐income country, the number of articles published in China is second only to Japan and the United States, and the number of articles published in China has been increasing in recent years. However, regarding total and average number of citations, IF, and other aspects, the quality of Chinese articles in this field must be improved. The keyword analysis shows that disease diagnosis, classification, autoantibodies, antibodies, prognosis and complications, and treatment are research hotspots in this field in recent years. Thus, we predict the anti mda5 antibody (anti‐mda5 antibody), mortality, gene 5 antibody, IIMs, double blind, and prognostic factors may be the new research hotspots. We hope that our research can provide information and support for understanding the relevant knowledge and research status in this field, and for predicting future hotspots to promote the continuous development of research in this field.

## 6 LIMITATIONS ANALYSIS

Although we thoroughly collated and analyzed the research on PM/DM with ILD, some shortcomings in the research process remained. First, our study was based only on the WoSCC database, and recollection from PubMed and Scopus databases is unknown. Second, both national economic level and English penetration may have some influence on our research results, though we have not conducted in‐depth research on the specific data for these two indicators. Our study was not sufficiently comprehensive in terms of data integrity and consistency. In addition, regarding retrieval time range, we limited the data collection period to 2000−2022. Bibliometric analysis was performed based on the accumulation of time, and the missing time period may contain some deviation from the complete and systematic research results in this field. Finally, two researchers (X. N. M. and W. F.) independently completed the collection and screening of articles and, afterwards, three researchers (X. N. M., W. F., and S. L. C.) discussed the inconsistent retrieval data and finally determined, sorted, and analyzed it. During this process, interference from human factors is inevitable which may influence the final analysis.

## AUTHOR CONTRIBUTIONS

**Xiao‐Na Ma, Wei Feng, Shu‐Lin Chen**: Data collection and collation, data analysis, visualization, methodology, manuscript writing. **Xiao‐Qin Zhong, Xue‐Xia Zheng**: Data verification, references, software. **Chang‐Song Lin**: Access to funding, resources. **Qiang Xu**: Conceptualization, funding acquisition, project management, resources, review and editing. Chang‐Song Lin and Qiang Xu are the sponsors of this study. Xiao‐Qin Zhong and Xue‐Xia Zheng also participated in this study. All authors read and approved the manuscript.

## CONFLICT OF INTEREST STATEMENT

The authors declare no conflict of interest.

## ETHICS STATEMENT

All the data in this study came from authoritative databases, and no human or animal experiments were conducted. Therefore, it does not require the approval of the Ethics Committee.

## Supporting information

Additional Figure 1: (A) Cooperation network among institutions in average year. (B) Cooperation network among authors in average year.

Additional Figure 2: Cluster analysis graph of co‐cited reference.

Additional Figure 3: (A) Co‐occurrence network visualization of keywords in average years. (B) The number of co‐occurrences of keywords ranked among the top 25 network visualizations.

Additional file 1‐4: Raw data related to this study.

Supporting information.

Supporting information.

Supporting information.

## Data Availability

The data that support the findings of this study are available from the corresponding author upon reasonable request.
