# Supplementary figures and images for: A bibliometric analysis of the research status and trends in studies on polymyositis and dermatomyositis with interstitial lung disease from 2000 to 2022 using Web of Science
Source: Immun Inflamm Dis. 2024 Feb 20;12(2):e1190. doi: 10.1002/iid3.1190 (PMC10878432; doi:10.1002/iid3.1190)

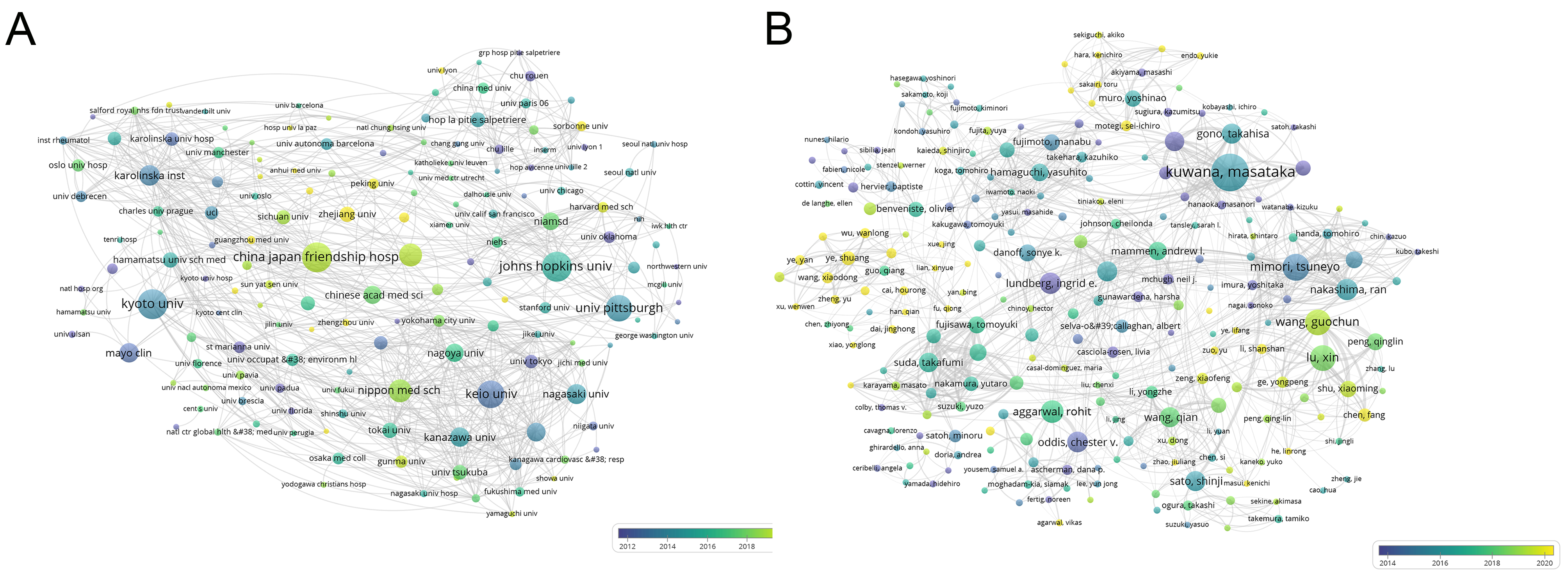

Supplement: Supplementary file 1 — Additional Figure 1: (A) Cooperation network among institutions in average year. (B) Cooperation network among authors in average year. [file IID3-12-e1190-s003.tif]

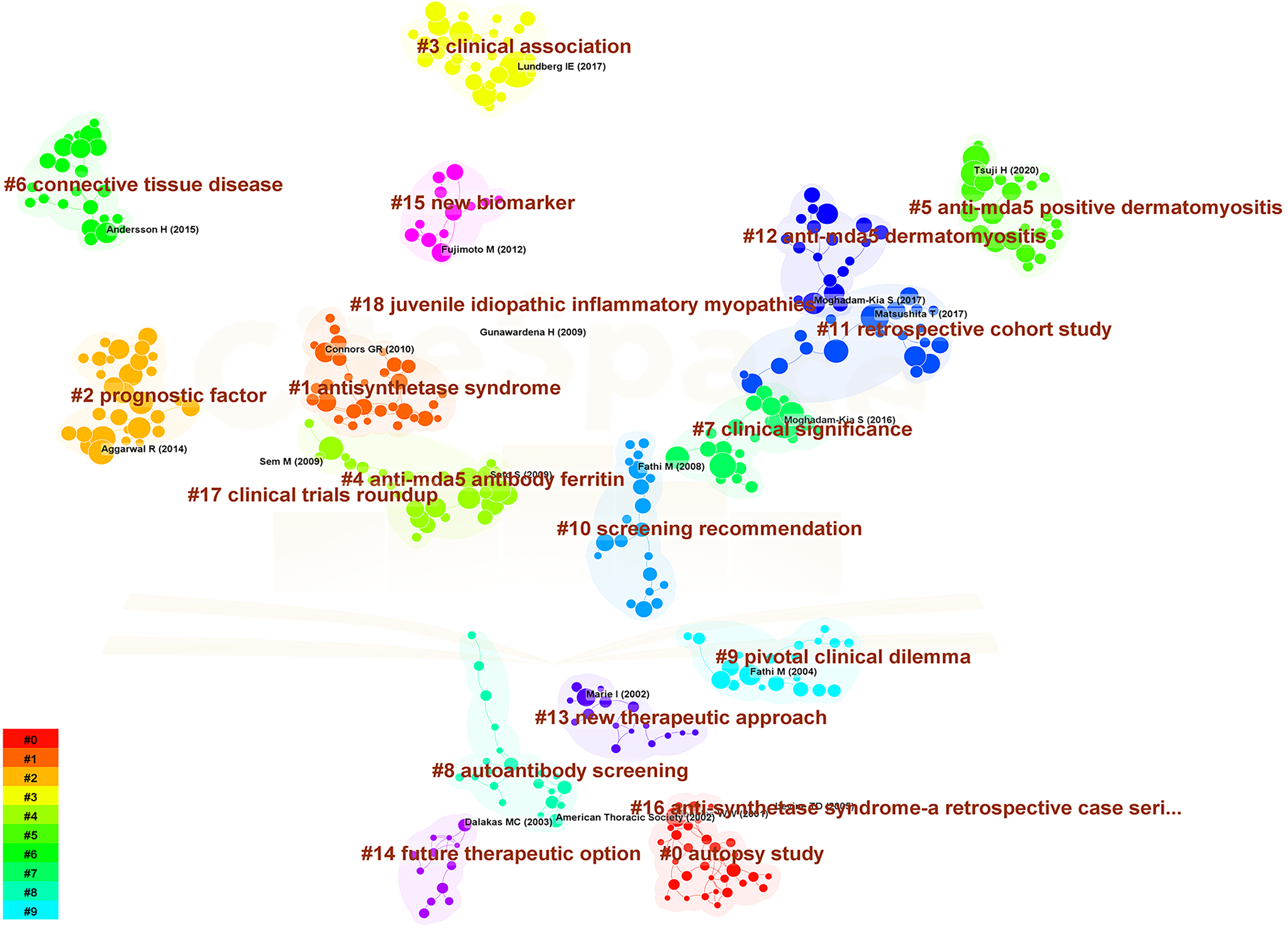

Supplement: Supplementary file 2 — Additional Figure 2: Cluster analysis graph of co‐cited reference. [file IID3-12-e1190-s005.tif]

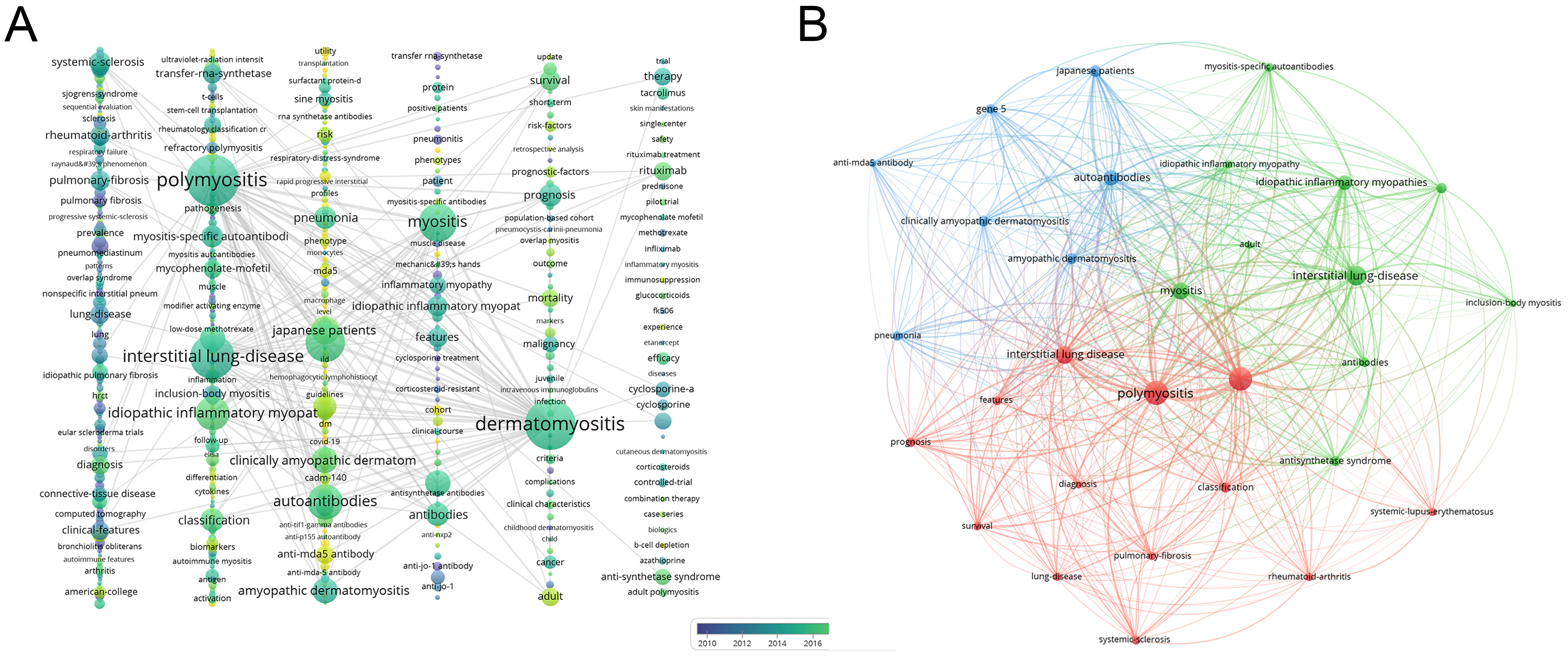

Supplement: Supplementary file 3 — Additional Figure 3: (A) Co‐occurrence network visualization of keywords in average years. (B) The number of co‐occurrences of keywords ranked among the top 25 network visualizations. [file IID3-12-e1190-s002.tif]
